# Emotional Cognition in Major Depressive, Bipolar and Borderline Personality Disorder - a Comparative Study of Recognition of Emotion in Auditory and Visual Stimuli

**DOI:** 10.1192/j.eurpsy.2026.11397

**Published:** 2026-07-31

**Authors:** J. J. Söderholm, L. Socada, T. Rosenström, J. Ekelund, E. Isometsä

**Affiliations:** 1 Department of Psychiatry, University of Helsinki; 2 Department of Psychiatry, HUS Helsinki University Hospital; 3 Department of Psychology, University of Helsinki, Helsinki, Finland

## Abstract

**Introduction:**

Negative cognitive biases, such as difficulties in recognizing emotions other than sadness, are a feature of major depressive disorder (MDD). Difficulties in recognition of emotional prosody (the emotional non-verbal content of speech) have also been found in MDD. They are postulated to be important for pathogenesis and recovery.

Major depressive episodes are also a feature of bipolar disorder (BD). Findings of emotional recognition of facial expressions in BD have been heterogenous, whereas recognition of prosodic information has been found to be impaired. However, direct comparisons of emotional cognition in depressed MDD and BD patients have been scarce.

Borderline personality disorder (BPD) frequently co-occurs with MDD and BD. How a MDE influences emotional cognition (EC) in BPD, and whether depressed persons with and without BPD differ from each other in this regard, is largely unknown.

Previous studies of EC have used varying analytical approaches. Many of these are based on unproven assumptions regarding the underlying distributions of cognitive processes. Regression analysis based on signal detection theory provides a flexible tool for overcoming some of these problems.

**Objectives:**

Our aim was studying EC in depressed patients with MDD, BD or BPD.

**Methods:**

This is a naturalistic cohort study of depression patients (N = 117), divided into MDD (n = 47), BD (n = 42) and BPD (n = 28) subcohorts.

In the facial emotion recognition task, they were presented with facial emotional stimuli expressing six basic emotions at different intensities and asked to classify the emotional valence. In the prosodic emotional recognition task, participants were presented with emotionally valenced stimuli in the form of a common name (“Saara”) spoken with a particular emotional prosodic tone, which they were asked to classify.

In addition to traditional analysis, we are applying multi-level signal detection regression models (using the glmmTMB-package in R), taking depression severity and specific diagnoses (MDD, BD and BPD) into account, yielding data on sensitivity and bias.

**Results:**

Our results will contribute to the understanding of emotional cognitive biases in different diagnostic groups. The directly comparative setting allows us to see in which ways the EC of depression patients with different specific diagnoses are alike, and in which they differ. Using multilevel signal detection regression models allows us to bypass many potentially problematic assumptions underlying traditionally used more primitive models, which may explain some of the heterogeneity in earlier literature.

**Conclusions:**

Our study contributes to the understanding of emotional cognition in major depression. Applying signal detection theory to emotional cognition tasks may lead to more valid results.

**Disclosure of Interest:**

None Declared

